# Online Positive Affect Journaling in the Improvement of Mental Distress and Well-Being in General Medical Patients With Elevated Anxiety Symptoms: A Preliminary Randomized Controlled Trial

**DOI:** 10.2196/11290

**Published:** 2018-12-10

**Authors:** Joshua M Smyth, Jillian A Johnson, Brandon J Auer, Erik Lehman, Giampaolo Talamo, Christopher N Sciamanna

**Affiliations:** 1 Department of Biobehavioral Health The Pennsylvania State University University Park, PA United States; 2 Department of Medicine Penn State College of Medicine The Pennsylvania State University Hershey, PA United States; 3 Department of Public Health Sciences Penn State College of Medicine The Pennsylvania State University Hershey, PA United States

**Keywords:** adult, anxiety, depression, emotions, expressed emotion, internet, stress, psychological/physiopathology, surveys and questionnaires, treatment outcome, writing

## Abstract

**Background:**

Positive affect journaling (PAJ), an emotion-focused self-regulation intervention, has been associated with positive outcomes among medical populations. It may be adapted for Web-based dissemination to address a need for scalable, evidence-based psychosocial interventions among distressed patients with medical conditions.

**Objective:**

This study aimed to examine the impact of a 12-week Web-based PAJ intervention on psychological distress and quality of life in general medical patients.

**Methods:**

A total of 70 adults with various medical conditions and elevated anxiety symptoms were recruited from local clinics and randomly assigned to a Web-based PAJ intervention (n=35) or usual care (n=35). The intervention group completed 15-min Web-based PAJ sessions on 3 days each week for 12 weeks. At baseline and the end of months 1 through 3, surveys of psychological, interpersonal, and physical well-being were completed.

**Results:**

Patients evidenced moderate sustained adherence to Web-based intervention. PAJ was associated with decreased mental distress and increased well-being relative to baseline. PAJ was also associated with less depressive symptoms and anxiety after 1 month and greater resilience after the first and second month, relative to usual care.

**Conclusions:**

Web-based PAJ may serve as an effective intervention for mitigating mental distress, increasing well-being, and enhancing physical functioning among medical populations. PAJ may be integrated into routine medical care to improve quality of life.

**Trial Registration:**

ClinicalTrials.gov NCT01873599; https://clinicaltrials.gov/ct2/show/NCT01873599 (Archived by WebCite at http://www.webcitation.org/73ZGFzD2Z)

## Introduction

### Background

At present, 60% of all people living in the United States have at least one chronic health condition, and 42% have multiple chronic conditions [1]. As advances in the treatment of disease continue to prolong life and the overall population continues to age, these numbers are likely to increase. The significant costs associated with managing medical conditions are well known. The majority of diseases hold the potential to worsen the overall health of patients by limiting their functional capacity, productivity, and health-related quality of life and are a major contributor to health care expenditures [2-4]. Patients with medical conditions face several challenges and often need to modify life aspirations, daily routines, and employment. Although some patients experience periods of grieving and adjustment after a diagnosis, many others experience sustained distress that can further impact physical and mental health and quality of life [4]. Given the link between severe or chronic medical conditions and psychological distress, it is not surprising that comorbidity between medical and mental health conditions is the rule rather than the exception [5]. The 2001 to 2003 National Comorbidity Survey Replication, for example, found that more than 68% of adults with a mental health disorder reported having at least one general medical illness and that 29% of people with a medical condition also had a comorbid mental health problem [6,7]. Stressful life events often precede anxiety and mood disorders [8], and the accompanying psychological strain associated with the diagnosis of, and living with, a major medical illness places these patients at risk for comorbidity and worse health outcomes overall. Stress and dysphoric mood generally may worsen the prognosis and progression of disparate diseases [9,10] and is a major contributor to many of the leading causes of death in the United States such as cancer, coronary heart disease, respiratory disorders, and suicide, among others [11]. In light of evidence that stress and dysphoria might be a modifiable risk factor for the development and progression of medical illnesses, finding ways to reduce distress in patients with one or more existing medical conditions is a major public health concern.

Psychological interventions (eg, cognitive behavioral therapy, CBT) have been shown to reduce psychological distress in chronic disease populations [12-14]. Although psychological interventions are increasingly desirable among patients [15], there are several barriers to accessing face-to-face psychological care among people with chronic health conditions (eg, cost or insurance coverage, access, and stigma) [15-18]. The internet has emerged as an effective tool for disseminating efficacious mental health interventions [19] and may serve to overcome some of these barriers to accessing mental health services. For example, a meta-analysis of internet-based CBT interventions observed that they are effective for reducing depression and anxiety [20], and a study by Farrer et al [21] observed a 44% reduction in depressive symptoms over 6 months among those randomized to internet-based CBT versus only 11% among controls. To date, however, these evidence-based internet interventions are either not readily accessible or widely disseminated among the general population and, therefore, do not address the problem of access to psychological services.

Relative to internet-based therapeutic or counseling interventions, positive affect journaling (PAJ), a simple intervention that is cost-efficient and easily disseminated to patients, is becoming increasingly popular. PAJ is a modified version of the traditional expressive writing paradigm [22,23] wherein the participants write about a traumatic experience for approximately 15- to 20-min intervals, often across a period of 3 to 5 days. Reviews of expressive writing suggested that it was modestly effective in improving a number of physical and mental health outcomes [24,25] although large heterogeneities in efficacy have been documented.

For example, several studies have found clinical benefits tied to expressive writing in patients with autoimmune and inflammatory conditions such as arthritic conditions, lupus, and asthma [25-29], fibromyalgia [30,31], irritable bowel syndrome [32], and HIV or AIDS [33,34]. In addition, expressive writing has been found to have beneficial effects on blood pressure [35] and on several health-relevant outcomes following the experience of a heart attack such as reduced numbers of medical appointments and prescription medications, increased self-care behaviors, improved cardiac symptoms [36], and improved health-related quality of life [37]. Expressive writing has also been associated with small, but consistent, improvements to well-being among diverse cancer groups—especially breast, renal, and prostate cancer patients [38]. Finally, a relatively small study of 40 people diagnosed with major depressive disorder found that those writing about their deepest thoughts and feelings related to emotional events had significant reductions in depression immediately after writing and over 1 month thereafter [39].

A number of efforts have been made to modify the original expressive writing approach to be better suited for use across several contexts and populations. One stream of this process is reflected in the integration of positive psychology, a large and growing area of research that has linked positive psychological and emotional dispositions and states of being (eg, optimism, happiness, subjective well-being, and positive affect) to various beneficial outcomes. Some of the reported benefits of these positive dispositions include fewer physical symptoms [40], faster wound healing [41], healthier functioning biological processes (eg, neuroendocrine, inflammatory, and cardiovascular activity) [42], better interpersonal relationships [43], higher quality of life [44], increased longevity [45], and decreased morbidity [46,47]. As such, the expressive writing paradigm has been adapted to have participants write about positive aspects of their lives and themselves (eg, making meaning out of or finding benefit in past experiences [48,49] and focusing on positive aspects of one’s self [50]) under the notion that this would yield similar benefits to those observed in the positive psychology literature. As a whole, we refer to this array of positive-focused writing approaches as PAJ.

Positive affect interventions among both patients and healthy individuals have led to improvements in a number of health outcomes. In 2 studies comparing an educational control (ie, educational workbook and behavioral contract) with a positive affect intervention (ie, self-affirmation inducement over bimonthly telephone sessions with staff and unexpected gifts before calls), positive affect improved medication adherence in hypertensive African American patients [51] and physical activity in patients following a percutaneous coronary procedure [52]. In addition, Stanton et al [53] found that 4 sessions of written expressive disclosure or benefit finding resulted in lower physical symptom reports and medical appointments among breast cancer patients at 3-month follow-up. In healthy samples, Armitage et al [54] found beneficial effects of completing a self-affirmation questionnaire or self-affirming implementation intention on alcohol intake at 1-month follow-up, whereas Burton and King [55] observed that participants randomized to write only 2 min for 2 consecutive days in a laboratory about a recent positive event showed moderate reductions in physical symptoms (Cohen *d*=0.65) at 4- to 6-week follow-up.

### Objectives

The goal of this randomized controlled trial was to examine whether a 12-week internet-based PAJ intervention could reduce mental distress (primary outcome) and positively influence psychological, interpersonal, and physical well-being (secondary outcomes), relative to usual care, in a heterogeneous sample of patients with elevated anxiety symptoms. It was hypothesized that participants randomized to the intervention would experience decreases in mental distress (ie, Hospital Anxiety and Depression Scale score; HADS) and improvements in psychological well-being (eg, perceived stress and resilience), interpersonal well-being (ie, social support), and physical well-being (eg, days during which pain inhibited usual activities) over the 12-week intervention period. It was also hypothesized that participants randomized to receive the intervention would report less mental distress and greater levels of psychological, interpersonal, and physical well-being than those in the control condition at each assessment period.

## Methods

### Sample and Recruitment

All study procedures were approved by the Pennsylvania State Hershey Medical Center’s (PSHMC) institutional review board, and all participants provided written informed consent before engaging in any research-related activity. This study was registered on ClinicalTrials.gov (reference number NCT01873599), with recruitment and active intervention occurring from June 2013 to February 2014.

Potential participants were recruited through flyers placed around the PSHMC campus and advertisements placed in PSHMC media and local community newspapers in central Pennsylvania. In addition, oncology patients at The Pennsylvania State University Hershey Cancer Institute with an Eastern Cooperative Oncology Group (ECOG) Performance Status score of 0 to 3 (not completely disabled) were identified through registry review and sent a letter describing the study. Participants were provided with a toll-free number to call if they were interested in participating, as well as an opt-out card that could be mailed back by those who were uninterested. Individuals who did not respond were contacted through phone by a research staff member within 2 weeks to determine their interest in participating.

Eligibility for inclusion was based on (1) English fluency, (2) between 21 and 80 years of age, (3) internet access, (4) self-report of moderate to significant stress during the last month, (5) not currently pregnant and no plans to become pregnant within the next 3 months, (6) no plans to move within the next 6 months, (7) no hospitalization for a psychiatric condition in the last year, (8) not a high risk for suicidality as assessed by selected questions from the Structured Clinical Interview for Diagnostic and Statistical Manual of Mental Disorders [56]. Although not an explicit requirement, it was assumed that potential participants be familiar with using a computer and accessing websites.

Individuals interested in participation and who met the initial inclusion criteria were invited for a laboratory visit and further assessed for eligibility. Eligible participants: (1) reported a score of 8 to 15 on the anxiety subscale of the HADS [57] and (2) had an ECOG performance status of 0 (fully active) through 3 (limited self-care) [58]. Participants who met all inclusion criteria were invited to participate.

### Random Assignment

**Figure 1:**
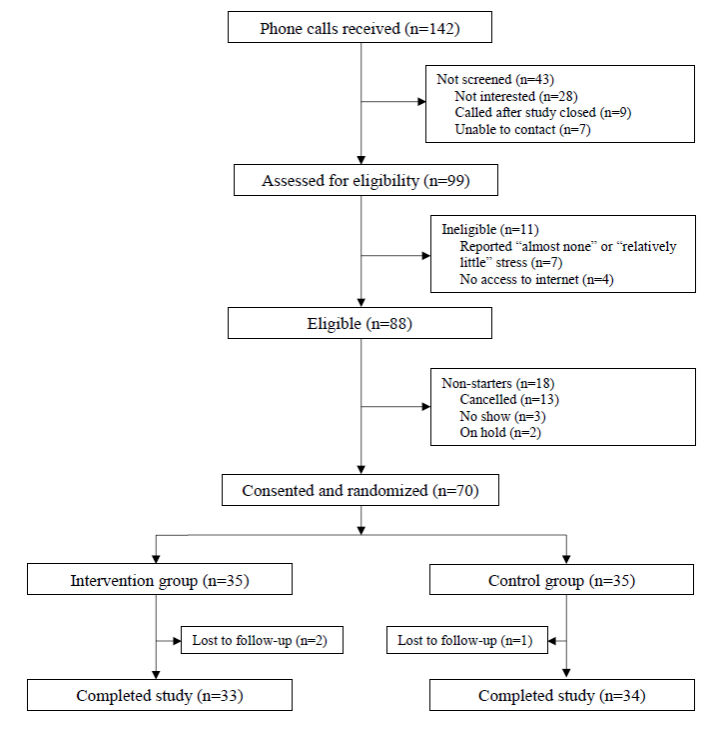
Participant flowchart.

Randomization (1:1) was done through sealed envelopes prepared by someone other than the research staff conducting the study visits and opened by participants during the baseline visit after completing informed consent. See Figure 1 for flow diagram of recruitment procedure.

### Procedure

Eligible participants met the research staff during a scheduled baseline visit to discuss study procedures and provide written informed consent. During the baseline visit, all participants completed baseline surveys and were randomized (through computer-generated sequences provided in sealed envelopes) to 1 of the 2 conditions. Participants assigned to the intervention condition received an introduction and training session to orient them to the intervention website where they would complete the writing sessions. All participants completed self-report survey assessments on the Web at the end of months 1, 2, and 3 using a secure data capture system (REDCap Penn State). Participants received gift cards following the completion of each survey (ie, US $40 compensation for completing all 3 assessments).

### Intervention

Participants in the PAJ intervention condition were asked to complete Web-based writing sessions for 15 min on 3 days each week for the duration of the 12-week study. The amount of time spent writing at each session is similar to prior expressive writing studies although the duration of the intervention in this study was longer than many other prior studies [23,25,59] to ensure the potential for adequate *dose* of intervention. This was the first version tested of this intervention; the intervention content was *frozen* during the trial and not adjusted. During each Web-based writing session, participants logged onto the study website and wrote a journal entry on 1 of the 7 commonly used positive affect prompts (eg, What are you thankful for? What did someone else do for you?; full details available upon request) [60]; all entries were saved on a secure server. During the study, journal entries of participants in the intervention condition were screened by research staff to monitor content. Participants who did not complete a journal entry within any given 7-day period were sent an email reminder (this email reminder also included reminders of how study staff could help them resolve any technical difficulties, in case, any existed). As there is no clinical standard of care treatment for medical patients with mild to moderate anxiety symptoms, participants randomized to the wait-list control group received their usual care for the duration of the study. After they had completed all study procedures, participants in the control condition were given access to the PAJ intervention.

### Measures

#### Sociodemographics and Health Behaviors

Participants’ age, gender, race, ethnicity, marital status, and educational level were obtained at baseline. During this time, participants self-reported basic information related to their specific disease and health behaviors (ie, smoking, physical activity, and alcohol use) using standard self-report items from the Behavioral Risk Factor Surveillance System [61].

#### Primary Outcome

The HADS [57] consists of 2 scales, anxiety and depression, with each consisting of 7 items rated on a scale from 0 through 3. Items are aggregated for each subscale (range=0-21), with higher scores indicating greater anxiety or depressive symptom severity, and for a total HADS score (range=0-42), with higher scores indicating greater mental distress. Various cut-off scores are available for the HADS. A score of 8 or greater on the anxiety subscale (HADS-A) has a specificity of 0.78 and sensitivity of 0.9 for clinically significant anxiety, whereas scores below 8 indicate noncases [62]; the inclusion criterion of a HADS-A score of 8 to 15 was intended to include participants with mild to moderate symptoms, whereas those with nonsignificant or severe symptoms (HADS-A scores of 15-21) were excluded as the PAJ intervention was expected to have limited benefit for those individuals. In this study, Cronbach alpha at baseline was .65 for anxiety, .86 for depression, and .85 for HADS total score.

#### Secondary Outcomes

The Brief Resilience Scale (BRS) [63] is a 6-item measure of perceived resilience, including items such as “It does not take me long to recover from a stressful event.” Each item is rated on a scale from 1 (strongly disagree) to 5 (strongly agree). All items are aggregated for a total score (range=6-30); higher scores indicate greater resilience. The BRS has high levels of internal consistency, with Cronbach alpha ranging from .80 to .91 [63]. In this study, Cronbach alpha was .90 at baseline.

The Healthy Days Measure [61] assesses self-reported physical health and functioning. Respondents answered 4 items to indicate (1) general health (ie, “Would you say that in general your health is ‘poor’ – ‘excellent’”), (2) days during which pain inhibited their usual activities (ie, “During the past 30 days, for about how many days did pain make it hard for you to do your usual activities...?”), (3) sleep quality (ie, “During the past 30 days, for about how many days have you felt you did not get enough rest or sleep?”), and (4) number of days they felt healthy and full of energy (ie, “During the past 30 days, for about how many days have you felt very healthy and full of energy?”).

The Perceived Stress Scale [64] consists of 10 items that assess perceived stress rated on a scale from 0 (never) to 4 (very often). A sample item includes “In the last month, how often have you felt nervous and stressed?” Items are combined for a total score, with higher scores indicating greater stress. The measure demonstrates strong internal consistency. In this study, Cronbach alpha was .91 at baseline.

The Positive and Negative Affect Schedule (PANAS) [65] consists of 2 subscales, each including 10 items. Respondents indicate the extent to which they felt specific positive emotions (eg, excited and proud) and negative emotions (eg, upset and afraid) over the last month on a scale from 1 (not at all) to 5 (extremely). Subscales are scored separately (range=10-50); higher scores indicate greater positive affect and greater negative affect. Internal consistencies are high (Cronbach alpha=.85-.88) [65]. In this study, Cronbach alpha for the positive affect subscale was .90 and negative affect subscale was .90 at baseline.

The Satisfaction with Life Scale (SWLS) [66] is a 5-item scale that assesses overall life satisfaction with items such as “In most ways my life is close to my ideal” and “I am satisfied with my life.” Each item is rated on a scale from 1 (strongly disagree) to 7 (strongly agree), and a total score is calculated using all items (range=5-35); higher scores indicate greater satisfaction with one’s life. The SWLS has a test-retest reliability of 0.82 [66]. In this study, Cronbach alpha was .93 at baseline.

The Social Provisions Scale [67] assesses various dimensions of social support. The scale consists of 24 items that make up 6 components, each consisting of 4 items pertaining to attachment, social integration, reassurance of worth, reliable alliance, guidance, and opportunity for nurturance. The respondent rates the extent to which each statement describes their current social network on a scale from 1 (strongly disagree) to 4 (strongly agree). Items are aggregated separately for each component (range=4-16) and for a total perceived support score (range=24-96); higher scores indicate a greater degree of perceived support provisions. In this study, Cronbach alpha for attachment was .76, .88 for social integration, .72 for reassurance of worth, .84 for reliable alliance, .83 for guidance, .79 for opportunity for nurturance, and .93 for total perceived support at baseline.

### Adherence

Adherence generally describes the extent to which individuals are exposed to the content of the intervention. For this study, participants were asked to complete Web-based PAJ sessions an average of three 15-min sessions per week, over 12 weeks, for a total of 36 journaling sessions throughout the course of the study. Overall PAJ adherence rate was calculated using 2 methods: (1) weekly journaling counts for each participant—derived from Web-based user log-in counts—were recoded into a binary variable (ie, yes or no) based on journaling >1 time per week. The journaling counts for all weeks were then summed, divided by 12, and multiplied by 100 to calculate the overall 12-week adherence rate and (2) total journaling counts for all participants were summed for all weeks of the study, divided by 36, and multiplied by 100. Although it would be desirable to count actual time (minutes per session) spent engaged in the PAJ intervention, the website was not capable of accurately tracking this information (eg, if a person left the computer to complete another task).

### Sample Size

The sample size was calculated based on an anticipated baseline mean of 11.0 (SD 3) on the HADS-A. This anticipated value was derived from a study of 273 medical patients participating in a Web-based educational program. Using G*Power, we assumed treatment condition SDs similar to those reported by Yun et al [68] and a 5% type-I error rate for a two-sided hypothesis test, concluding that 31 subjects per group would provide 80% power to detect a difference in the HADS-A at 3 months (10.0 vs 8.0). This effect size is based on a clinical trial of CBT for distressed medical patients wherein the CBT arm decreased their HADS-A score by 3.1 points more than controls [7], and a Web-based CBT intervention by Farrer et al [21] observed a 44% reduction in depression scores over 6 months. This study estimated a reduction of 2.0 in the HADS-A measure (18% reduction). Anticipating a dropout rate of <10%, we planned to recruit 70 subjects at baseline.

### Analytic Plan

All analyses were conducted using SAS Software version 9.4 (SAS Institute, Cary, NC). First, descriptive statistics were calculated for all variables at baseline and at each of the 3 follow-up assessments, and response rates were calculated using the Web-based user log-in tracking logs. Categorical variables were summarized with frequencies and percentages, and continuous variables were summarized with means, SDs, medians, and quartiles. The distribution of continuous variables was checked using box plots, histograms, and normal probability plots. For demographic variables and other characteristics measured at baseline, comparison tests were conducted between the intervention and control groups using a two-sample *t* test or Wilcoxon rank-sum test with means for continuous variables and using a chi-square test with percentages for categorical variables. A Fisher exact test was used as needed when cell counts were too small for the chi-square test to be valid.

Second, in making comparisons of the differences from baseline to each of the 3 months within and between groups, we used 2 approaches depending on the type of outcome variable. For continuous outcome variables, we first found the change from baseline at each subsequent month. A linear mixed-effects model was then employed, which included factors for group (intervention vs control), month, the interaction between the intervention group and month, and the baseline measurement for adjustment, and the differences between groups were quantified with means. For binary outcome variables, a generalized estimating equations model was used that included factors for group, month, and the interaction between the group and month, and differences between groups were quantified with percentages and odds ratios. All comparisons were adjusted for age, sex, income, and preexisting journaling—reflecting self-reported frequency (ie, “Never,” “Less than once per month,” “1-3 times per month,” and “At least once per week”) of writing in a diary or journal in the year leading up to the study—by including these factors as additional covariates in the models. Missing data were not a significant problem for the primary outcome variable (at less than 5%) or for the secondary outcome variables (at less than 10% at most) and were not an issue for any independent variables.

## Results

### Participants

A total of 99 people were assessed for eligibility, of which 88 patients were interested in participating. After further screening, 70 people were eligible, consented, and randomized to the intervention (n=35) or usual care (n=35) condition (Figure 1). A total of 3 participants were lost to follow-up during the 12-week assessment period and all participants were included in analyses. No unanticipated harms were reported, and there were no privacy breaches or major technical problems during the trial. Participants in this study had a broad range of chronic health conditions, including arthritic conditions (eg, rheumatoid arthritis, gout, lupus, and fibromyalgia; 19/69, 27%), diabetes (type 1 or type 2; 12/70, 17%), asthma (12/70, 17%), cancer (13/70, 11%-19%, all cancer types combined), prediabetes (4/70, 6%), kidney disease (not including kidney stones, bladder infection, or incontinence; 3/69, 4%), chronic obstructive pulmonary disease (1/69, 1%), heart disease (1/70, 1%), and stroke (1/69, 1%). Demographic and other baseline characteristics are shown in Table 1. There were no significant differences between the intervention and control groups on any baseline characteristics (demographics, primary, or secondary outcomes).

### Within-Group Differences

Our initial analyses examined changes over time within each group. All results for the within-group differences across the 3-month study period are shown in Multimedia Appendix 1. Results indicated that the PAJ intervention reduced mental distress and improved well-being. Specifically, the intervention group reported lower HADS-A at all 3 assessments (at the end of months 1 through 3), more resilience at the end of month 2, less perceived stress at all 3 assessment points, and a greater percentage (ie, 56.3% vs 31.3%) of participants reported better mental health at the end of the first month, relative to baseline. No other within-group differences were observed in the intervention group. Compared with the baseline, the control group reported less social integration at the end of month 3, more days in pain inhibiting usual activities at the end of month 2, and a greater percentage (ie, 41.4% vs 20%) of participants reported better mental health at the end of month 3, relative to the previous month. No other within-group differences were observed in the control group.

### Between-Group Differences

We next examined differences between the groups over time. Results for the between-group differences across the 3-month study period are also indicated in Multimedia Appendix 1. Compared with the control group, the intervention group exhibited lower anxiety at the end of month 1, lower mental distress at the end of months 1 and 2, greater resilience and lower perceived stress at the end of month 1, greater self-reported social integration at the end of month 2, and at the end of month 2, they reported fewer days in which pain prohibited usual activities.

### Adherence

**Figure 2:**
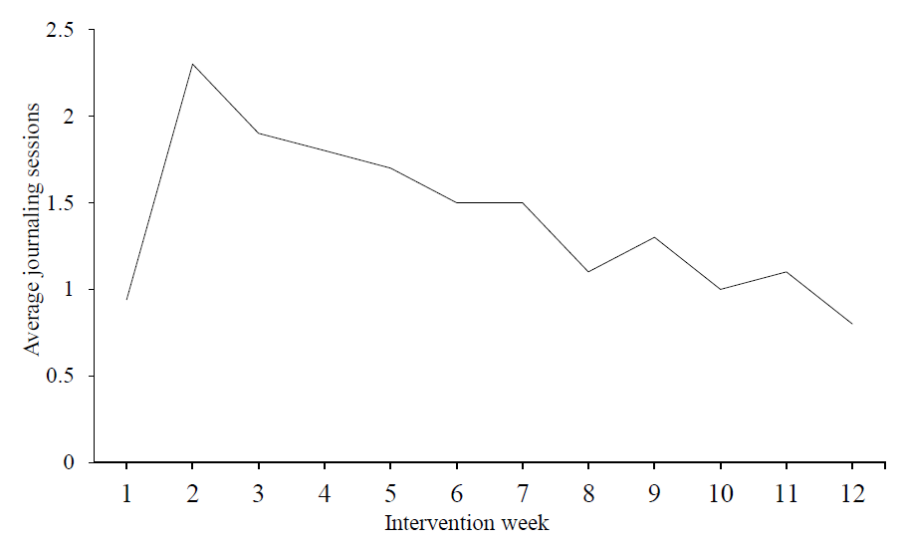
Average number of journaling sessions completed by participants over the 12-week study period.

We also examined patient adherence to suggested journaling frequency (or *dose*). Overall adherence to the intervention, operationalized by dividing the mean amount of completed sessions by the maximum amount of sessions, was moderate in the sample from this study (mean 47.8% with a range of 2.8%-172.2%; 1 participant journaled 62 times with the remainder having rates at or below the expected 100%). When operationalizing adherence as completing at least one journaling session per week, a level consistent with the broader expressive writing literature [23], the adherence rate was 66.4% (range of 41.7%-100%). After the first week of journaling, participants journaled an average of 0.94 times with a peak of 2.3 times per week in week 2. Overall, the number of journaling sessions generally decreased as time progressed (see Figure 2). Adherence to PAJ sessions was largely unrelated to outcomes (data not shown; results available upon request).

**Table 1:** Participant and baseline characteristics.

Characteristic		Total (N=70)	Control (n=35)	Intervention (n=35)	*P* value^a^
Age in years, mean (SD)		46.9 (12.8)	47.2 (12.3)	46.5 (13.5)	.82
Female, n (%)		60 (87)	30 (85)	30 (88)	.99
White, n (%)		65 (95)	33 (94)	32 (97)	.99
Hispanic, n (%)		1 (1)	1 (3)	0 (0)	.99
Married, n (%)		44 (64)	25 (71)	19 (56)	.18
Education (college 4+ years), n (%)		41 (59)	18 (51)	23 (68)	.17
Employed for wages, n (%)		53 (77)	26 (74)	27 (79)	.61
Income (<US $50,000), n (%)		21 (33)	9 (30)	12 (35)	.65
Current smoker, n (%)		3 (4)	1 (3)	2 (6)	.99
General health (excellent or very good), n (%)		34 (49)	14 (41)	20 (57)	.19
**Hospital anxiety and depression scale, mean (SD)**					
	Total	14.3 (6.6)	14.3 (7.1)	14.3 (6.1)	.74
	Anxiety	9.8 (3.4)	9.5 (3.4)	10.1 (3.4)	.44
	Depression	4.6 (4.0)	4.9 (4.2)	4.3 (3.7)	.68
Perceived stress scale, mean (SD)		19.9 (7.2)	20.4 (6.7)	19.4 (7.7)	.55
Brief resilience scale, mean (SD)		20.1 (5.3)	20.8 (4.3)	19.5 (6.1)	.37
Satisfaction with life scale, mean (SD)		19.0 (8.0)	19.7 (7.3)	18.3 (8.6)	.48
**Social provisions scale, mean (SD)**					
	Total	79.0 (12.0)	78.6 (10.9)	79.3 (13.1)	.55
	Attachment	12.5 (2.6)	12.6 (2.4)	12.5 (2.8)	.94
	Social integration	13.2 (2.7)	13.3 (2.4)	13.1 (2.9)	.94
	Reassurance of worth	12.9 (2.2)	12.8 (1.9)	12.9 (2.4)	.41
	Reliable alliance	13.6 (2.6)	13.4 (2.6)	13.8 (2.6)	.47
	Guidance	13.3 (2.7)	13.1 (2.6)	13.5 (2.9)	.35
	Opportunity for nurturance	13.6 (2.5)	13.7 (2.2)	13.5 (2.7)	.96
**Positive and negative affect scale, mean (SD)**					
	Positive affect	31.9 (8.2)	33.4 (7.8)	30.4 (8.4)	.15
	Negative effect	16.0 (6.6)	16.7 (6.9)	15.3 (6.2)	.20
Days pain inhibited usual activities, mean (SD)		3.5 (6.9)	3.7 (6.1)	3.4 (7.6)	.08
Days not getting enough sleep, mean (SD)		12.3 (9.7)	13.1 (10.3)	11.5 (9.2)	.68
Days felt healthy and full of energy, mean (SD)		14.0 (10.0)	12.8 (9.4)	15.3 (10.5)	.30
Better mental health than 1 month ago, n (%)		17 (25)	6 (18)	11 (31)	.18

^a^*P* values for variables based on means are from a two-sample *t* test or Wilcoxon rank-sum test; *P* values for variables based on percentages are from a chi-square test; Fisher exact tests were used as needed.

## Discussion

### Principal Findings

The primary aim of this study was to examine whether a 12-week Web-based PAJ intervention could reduce mental distress and improve psychological, interpersonal, and physical well-being in a heterogeneous sample of medical patients with significant anxiety symptoms. Compared with the patients receiving standard care, patients randomized to the PAJ intervention exhibited reduced mental distress, anxiety, and perceived stress; greater perceived personal resilience and social integration; and fewer days on which pain inhibited usual activities. The PAJ intervention was not associated with improvements in depressive symptoms, satisfaction with life, other indices of social support (ie, attachment, reassurance of worth, reliable alliance, guidance, opportunity for nurturance, and overall perceived support), or positive and negative affect. Overall, the findings from this study suggest that PAJ has potential utility as an intervention for managing mental distress, particularly elevated anxiety symptoms, and other aspects of well-being among general medical patients. This is consistent with, and extends, prior research on positive writing interventions as a way to improve aspects of health and well-being [55,69,70,71].

### Effects on Well-Being

This study demonstrates that PAJ can improve several factors associated with psychological well-being among patients with mild to moderate anxiety, each of which may have implications for long-term health outcomes. Some of the most notable findings from this study were that the PAJ intervention was associated with better mental health, including lower anxiety, mental distress, and perceived stress after only 1 month of the intervention and was associated with reduced mental distress across time (eg, continued reduction in anxiety and perceived stress across the 12-week intervention period). As such, PAJ may be an effective way to improve mental well-being and potentially increase longevity through improvements in these outcomes in a variety of patient populations. In addition, PAJ was associated with higher perceived resilience. Although PAJ may be a useful intervention for improving this outcome, more work is needed to understand why the beneficial effects of PAJ on resiliency appear to taper off over time and whether the initial increase in resiliency may serve as a mechanistic pathway between PAJ and disease outcomes across a variety of medical conditions.

Another aspect of psychological well-being with potential implications for health outcomes are perceptions of one’s social environment such as perceived social integration. For example, perceived social isolation, which is conceptually the opposite of perceived social integration, is known to contribute to increased risk for early mortality [72]. This study indicates that PAJ may be beneficial to this end as the results demonstrate greater self-reported social integration in the intervention group relative to controls at the end of the second month. Moreover, more work is needed to understand why the effects of PAJ on perceived social integration do not appear to hold over time and whether these benefits can be prolonged.

Surprisingly, 1 indicator of improved well-being (ie, percent of patients who self-reported “somewhat” or “much better” mental health compared with the previous month) was observed in the control group. This indicates that patients receiving usual care will fluctuate in their perceived well-being (ie, have occasional upswings in self-reported mental health). In addition, PAJ was not observed to improve all indices of well-being (ie, we did not see beneficial effects on depression, satisfaction with life, indices of social support other than perceived integration, or positive and negative affect). This indicates that this intervention may be effective for improving some but certainly not all aspects of well-being. To explore the robustness of PAJ for enhancing additional indices of well-being, future studies would benefit from exploring ways to modify the expressive writing methods used in this study to increase their effectiveness (eg, across more well-being outcomes, for longer durations of time, or both). For example, the writing schedule in this study was fairly dense (3 writing sessions per week for 12 weeks). Perhaps, patients would benefit from a less dense writing schedule or greater variability in the topics offered. It may also be possible to optimize benefit by tailoring PAJ instructions (overall or adaptively over time) to individual patient needs. Furthermore, the writing task used in this study was a modified version of that developed by Pennebaker et al [23], particularly in terms of the number of overall sessions; it is possible that writing in a manner more consistent with earlier expressive writing studies would be preferable.

### Timing Effects

Regarding the timing effects as a whole, the first month of the PAJ intervention provides a considerable number of improvements in quality of life that are still observed 2 and 3 months later, albeit to a lesser degree. For example, PAJ was associated with decreased mental distress and improved well-being in the first month, but the number of benefits and between-group differences diminished over time. Perhaps, the benefits of PAJ are largely observed within only at the start of the intervention and do not provide sustained improvements in mental distress and well-being over time. However, other studies have demonstrated that longer-lasting positive psychology interventions are effective at improving subjective and psychological well-being among cancer patients [73], and the *short-term* improvements in cancer patients’ mental well-being observed in this study may translate into longer-term health benefits. As such, future studies are needed to determine whether PAJ beyond 3 months would provide additional upswings in well-being. Conducting longer investigations of expressive writing in clinical populations may be particularly important as previous work has found the benefits to dissipate after several months of discontinuation [28]. It is worth noting that the sample size of our study was modest, and even after just 3 months, improvements were observed that favor the PAJ group. Although a portion of the effect sizes for these significant improvements were small (ie, Cohen *d* or *h*: 0.5-0.49), there were also several effect sizes of moderate size (ie, Cohen *d* or *h*: 0.51-0.64; see Multimedia Appendix 1). Given the potential for cost-efficiency and reach of this Web-based intervention, we view these preliminary results as promising and supportive of a larger follow-up study examining the clinical utility of PAJ interventions.

### Feasibility of Intervention

An important aspect of this study is the demonstrated feasibility of the Web-based writing task intervention. First, participants generally enjoyed the intervention (ie, 39.4% reported that the journaling activity made them feel “somewhat better” and 18.2% reported that it made them feel “much better”). A total of 67 out of 70 consented and randomized participants competed the study for an overall excellent completion rate of 95%; this compares favorably with other randomized expressive writing interventions in chronic illness samples (eg, 73% [74] and 81% [53]). Overall adherence to the intervention was moderate in the sample of this study (mean 47.8%). However, when operationalizing adherence as completing at least one journaling session per week, the adherence rate rose to 66.4% (range of 41.7%-100%). Once-weekly sessions are common in therapeutic practice and are frequently used in randomized trials of CBT [75]. Although these adherence rates are acceptable, it remains unclear why adherence was not even higher, given the relative ease through which Web-based PAJ modules could be accessed.

Generally, the number of completed journaling sessions decreased over the course of the intervention. The reasons for this decrease are uncertain but several plausible explanations exist. One possibility, although unassessed in this study, is that participants began the journaling process with enthusiasm in the early weeks but experienced reduced interest or increased fatigue with the intervention over time. Another possible explanation is that participants believed there were diminishing returns on therapeutic benefit as journaling sessions increased; a single weekly journaling session could have been deemed therapeutically equivalent to multiple sessions, for example, Web-based interventions are becoming more prevalent [76] as they can be administered at lower costs and disseminated to more people. Future work should investigate factors that drive adherence to Web-based PAJ interventions and explore opportunities to improve the interventions themselves, given the benefits observed in this study.

### Limitations

Given the preliminary nature of investigating this novel Web-based intervention, we included several outcomes and conducted a large number of statistical tests, and the small sample size reduced our power to detect some effects (especially when contrasting between groups); together, these decisions may have contributed to spurious and/or missed associations. As such, care should be taken in interpreting any specific effect, and replication of these effects is warranted before strong conclusions can be made about potential efficacy. In addition, the length of the study was relatively short, and it remains unclear whether longer-term interventions would be sustainable or show similar improvements in various indices of well-being. Evidence from some clinical populations (eg, patients with asthma [29]) suggests that expressive writing may offer the most benefit for those with moderate level of disease—patients who are relatively healthy do not have much room for improvement, and those with very severe illness may require a more powerful treatment alternative. A large percentage (44.3%) of patients in this study reported excellent or very good health at baseline, possibly limiting the therapeutic benefit that could be observed from journaling; less healthy patients may have greater gains to make in well-being from baseline relative to their healthier counterparts, and future studies may consider testing this intervention in a sample of patients with greater disease severity. Finally, the homogenous nature of our small study sample (95.5% white and 87.0% female), combined with the relatively brief study time frame, limits the generalizability of our findings to more diverse patient samples.

### Conclusions

The results of this randomized controlled trial provide preliminary evidence that PAJ is a feasible and well-accepted intervention that can be implemented on the Web for effectively reducing some aspects of mental distress and improving aspects of well-being among medical patients with mild to moderate anxiety symptoms. Moreover, PAJ is likely to be a more pleasant and uplifting treatment for patients compared with the traditional expressive writing interventions that focus on writing about deeply distressing and traumatic experiences from the past; this may promote acceptability and treatment engagement relative to other treatments. Thus, this relatively simple and cost-effective intervention may represent a low-risk way to improve a variety of well-being domains, particularly among underserved patients.

## Appendix

Multimedia Appendix 1 Outcome variables over time.

Multimedia Appendix 2 CONSORT‐EHEALTH checklist (V 1.6.1).

## Abbreviations

BRS: Brief Resilience Scale
CBT: cognitive behavioral therapy
ECOG: Eastern Cooperative Oncology Group
HADS: Hospital Anxiety and Depression Scale
PAJ: positive affect journaling
PANAS: Positive and Negative Affect Schedule
PSHMC: Pennsylvania State Hershey Medical Center
SWLS: Satisfaction with Life Scale
