# Supplementary material for: Online Positive Affect Journaling in the Improvement of Mental Distress and Well-Being in General Medical Patients With Elevated Anxiety Symptoms: A Preliminary Randomized Controlled Trial
Source: JMIR Ment Health. 2018 Dec 10;5(4):e11290. doi: 10.2196/11290 (PMC6305886; doi:10.2196/11290)
Supplement: Multimedia Appendix 1 [file mental_v5i4e11290_app1.pdf]

Table 2. Outcome variables over time.

		Control (n=30)			Intervention (n=32)			Intervention vs. Control
Outcome	Time	Mean [95% CI] or %	*d* or *h*	*P*	Mean [95% CI] or %	*d* or *h*	*P*	*P*
**Hospital Anxiety and Depression Scale – total**								
	0	14.4 [11.7, 17.1]			13.8 [11.9, 15.7]			
	1	14.8 [12.2, 17.3]	.06	.44	11.4 [9.5, 13.2]	.48	.06	*.01*
	2	14.3 [11.5, 17.1]	.01	.72	11.5 [9.6, 13.5]	.43	.06	*.03*
	3	14.3 [11.4, 17.1]	.02	.92	12.2 [9.7, 14.6]	.27	.25	.27
**Hospital Anxiety and Depression Scale – anxiety**								
	0	9.6 [8.3, 10.9]			10.0 [8.8, 11.2]			
	1	9.7 [8.2, 11.1]	.01	.60	7.9 [6.8, 9.0]	.64	*<0.001*	*.01*
	2	9.1 [7.5, 10.6]	.14	.18	8.2 [7.0, 9.3]	.55	*0.002*	.09
	3	9.0 [7.5, 10.5]	.16	.14	8.3 [7.0, 9.6]	.49	*0.003*	.19
**Hospital Anxiety and Depression Scale – depression**								
	0	4.9 [3.3, 6.4]			3.8 [2.9, 4.8]			
	1	4.9 [3.3, 6.4]	.02	.22	3.4 [2.4, 4.4]	.14	.97	.19
	2	5.1 [3.7, 6.5]	.06	.14	3.4 [2.3, 4.4]	.16	.89	.11
	3	5.0 [3.5, 6.6]	.04	.32	3.9 [2.5, 5.2]	.02	.58	.69
**Brief Resilience Scale**								
	0	20.3 [18.7, 21.9]			19.7 [17.6, 21.8]			
	1	19.2 [17.2, 21.1]	.24	.82	20.7 [18.8, 22.6]	.17	.09	*.049*

	2	20.1 [18.3, 21.9]	.05	.50	21.5 [19.5, 23.6]	.31	*.01*	.06
	3	20.1 [17.9, 22.3]	.04	.43	20.9 [18.9, 22.8]	.20	.08	.35
**Perceived Stress Scale**								
	0	19.9 [17.4, 22.4]			19.0 [16.3, 21.7]			
	1	20.3 [17.7, 23.0]	.06	.93	16.9 [14.5, 19.3]	.30	*.03*	*.047*
	2	18.9 [16.6, 21.3]	.15	.27	16.6 [14.0, 19.3]	.32	*.02*	.24
	3	19.9 [17.1, 22.7]	.00	.48	17.0 [14.1, 19.9]	.26	*.04*	.17
**Satisfaction With Life Scale**								
	0	20.2 [17.4, 22.9]			18.8 [15.9, 21.7]			
	1	20.3 [17.4, 23.3]	.02	.78	19.8 [17.0, 22.5]	.12	.50	.69
	2	20.2 [17.1, 23.2]	.00	.88	20.2 [17.3, 23.0]	.18	.32	.41
	3	20.3 [17.1, 23.5]	.02	.73	19.6 [16.7, 22.5]	.10	.59	.86
**Social Provisions Scale – total**								
	0	79.0 [74.6, 83.3]			79.5 [74.8, 84.1]			
	1	78.1 [73.3, 82.8]	.08	.31	79.5 [75.2, 83.8]	.00	.73	.49
	2	78.2 [73.9, 82.5]	.07	.41	81.8 [77.1, 86.5]	.18	.48	.11
	3	78.1 [73.2, 82.9]	.08	.33	80.3 [75.8, 84.9]	.07	.98	.31
**Social Provisions Scale – attachment**								
	0	12.7 [11.7, 13.6]			12.5 [11.5, 13.5]			
	1	12.2 [11.1, 13.4]	.15	.47	12.7 [11.7, 13.8]	.08	.66	.29
	2	12.4 [11.5, 13.4]	.10	.68	12.9 [11.8, 13.9]	.13	.42	.22
	3	12.6 [11.6, 13.6]	.02	.89	13.1 [12.1, 14.1]	.20	.23	.18
**Social Provisions Scale - social integration**								

	0	13.3 [12.4, 14.2]			13.2 [12.1, 14.2]			
	1	12.7 [11.8, 13.6]	.26	.11	12.9 [12.0, 13.7]	.11	.40	.43
	2	12.9 [12.0, 13.7]	.20	.25	13.5 [12.6, 14.5]	.15	.37	*.04*
	3	12.4 [11.5, 13.2]	.42	*.03*	13.2 [12.3, 14.1]	.01	.93	.05
**Social Provisions Scale - reassurance of worth**								
	0	12.6 [11.9, 13.3]			13.2 [12.4, 13.9]			
	1	12.5 [11.6, 13.4]	.07	.15	13.2 [12.3, 14.0]	.00	.61	.35
	2	12.2 [11.4, 13.1]	.19	.09	13.6 [12.8, 14.4]	.20	.84	.05
	3	12.1 [11.2, 13.0]	.27	.07	13.2 [12.4, 14.0]	.01	.58	.21
**Social Provisions Scale - reliable alliance**								
	0	13.3 [12.3, 14.3]			13.6 [12.6, 14.5]			
	1	12.9 [11.7, 14.0]	.15	.20	13.9 [13.1, 14.7]	.13	.63	.08
	2	13.3 [12.4, 14.3]	.01	.79	14.3 [13.5, 15.2]	.30	.16	.08
	3	13.0 [11.9, 14.2]	.10	.48	13.6 [12.6, 14.5]	.00	.85	.61
**Social Provisions Scale – guidance**								
	0	13.1 [12.1, 14.0]			13.5 [12.4, 14.5]			
	1	13.2 [12.3, 14.1]	.07	.86	13.5 [12.5, 14.4]	.00	.97	.89
	2	13.0 [12.1, 13.9]	.03	.73	13.7 [12.6, 14.9]	.09	.60	.39
	3	12.7 [11.7, 13.8]	.13	.46	13.7 [12.8, 14.7]	.10	.52	.17
**Social Provisions Scale - opportunity for nurturance**								
	0	13.8 [13.1, 14.5]			13.5 [12.5, 14.4]			
	1	14.0 [13.2, 14.8]	.08	.13	13.1 [12.2, 14.0]	.15	.76	.09
	2	13.9 [13.2, 14.7]	.05	.15	13.4 [12.5, 14.3]	.03	.62	.35
	3	14.0 [13.2, 14.8]	.08	.07	13.5 [12.6, 14.5]	.02	.41	.33

**Positive and Negative Affect Scale - positive affect**								
	0	32.9 [30.1, 35.8]			30.6 [27.6, 33.6]			
	1	30.7 [27.7, 33.7]	.28	.44	27.9 [24.3, 31.5]	.29	.21	.65
	2	30.8 [27.5, 34.2]	.25	.46	29.2 [25.4, 33.0]	.14	.51	.91
	3	30.9 [27.6, 34.1]	.25	.52	28.0 [24.0, 32.1]	.26	.22	.59
**Positive and Negative Affect Scale - negative affect**								
	0	16.9 [14.1, 19.6]			14.9 [13.0, 16.8]			
	1	19.6 [16.4, 22.8]	.34	.14	15.8 [13.8, 17.7]	.16	.89	.17
	2	19.6 [16.8, 22.4]	.37	.15	17.3 [14.7, 19.8]	.38	.25	.75
	3	18.7 [15.4, 22.0]	.23	.42	16.0 [13.6, 18.5]	.19	.73	.61
**General health (%)**								
	0	45			59			
	1	35	.21	.31	59	.00	.99	.35
	2	30	.31	.14	53	.13	.14	.43
	3	35	.21	.45	47	.25	.21	.81
**Days pain inhibited usual activities**								
	0	4.0 [1.6, 6.4]			3.7 [0.8, 6.5]			
	1	4.9 [1.5, 8.2]	.11	.24	3.1 [0.3, 5.9]	.08	.95	.24
	2	6.2 [3.0, 9.3]	.29	*.02*	3.3 [0.6, 6.0]	.05	.93	*.02*
	3	3.9 [1.7, 6.0]	.02	.62	4.2 [1.0, 7.3]	.06	.49	.86
**Days not getting enough sleep**								
	0	13.8 [9.9, 17.7]			11.7 [8.3, 15.2]			
	1	13.4 [9.7, 17.1]	.05	.51	11.1 [7.9, 14.3]	.07	.19	.49

	2	14.0 [10.5, 17.4]	.01	.59	11.4 [8.2, 14.5]	.04	.23	.51
	3	13.6 [9.7, 17.4]	.03	.50	10.9 [7.6, 14.1]	.10	.19	.55
**Days felt healthy and full of energy**								
	0	13.2 [9.7, 16.7]			14.8 [11.0, 18.7]			
	1	12.9 [9.2, 16.5]	.04	.80	15.4 [11.6, 19.2]	.06	.38	.53
	2	13.9 [10.4, 17.3]	.07	.49	15.7 [12.1, 19.4]	.09	.50	.98
	3	15.1 [11.6, 18.5]	.21	.21	13.5 [10.0, 17.0]	.13	.91	.18
**Better mental health (%)**								
	0	20			31			
	1	27	.16	.47	56	.51	*.03*	.37
	2	30	.23	.36	44	.26	.32	.97
	3	41	.47	*.02*	41	.19	.37	.28

Note. *P* values are from a linear mixed effects model (means) with group, month, and interaction between month and group as factors in addition n to covariates for adjustment. Monthly means are raw means while change from baseline means are from the model adjusted for covariates. Significant *P* values are bold and italicized.
